# No significant association between self‐reported physical activity and brain volumes in women and men from five European cohorts

**DOI:** 10.1002/alz70860_097396

**Published:** 2025-12-23

**Authors:** Naiara Demnitz, William F. C. Baare, Julia Binnewies, Andreas Brandmaier, Anders M Fjell, Anne T Gates, Rogier A Kievit, Michael Kjaer, Kathrine Skak Madsen, Lars Nyberg, Sara Pudas, Hartwig R Siebner, Sana Suri, Øystein Sørensen, Kristine B Walhovd, Klaus P Ebmeier, Carl‐Johan Boraxbekk

**Affiliations:** ^1^ Copenhagen University Hospital ‐ Amager and Hvidovre, Hvidovre, Denmark; ^2^ Vrije Universiteit Amsterdam, Amsterdam, Netherlands; ^3^ MSB Medical School Berlin, Berlin, Germany; ^4^ Max Planck Institute for Human Development, Berlin, Germany; ^5^ University of Oslo, Oslo, Norway; ^6^ Oslo University Hospital, Oslo, Norway; ^7^ Copenhagen University Hospital ‐ Bispebjerg and Frederiksberg, Copenhagen, Denmark; ^8^ Radboud University Medical Center, Nijmegen, Netherlands; ^9^ University of Copenhagen, Copenhagen, Denmark; ^10^ Umeå University, Umeå, Sweden; ^11^ University of Oxford, Oxford, England, United Kingdom; ^12^ LCBC, University of Oslo, Oslo, Norway; ^13^ University of Oxford, Oxford, Oxford, United Kingdom; ^14^ Copenhagen University Hospital Frederiksberg and Bispebjerg, Copenhagen, Denmark

## Abstract

**Background:**

Various studies have reported an association between physical activity and grey matter volumes. Some studies have suggested that this relationship may be moderated by sex, yet the direction is still under debate.

**Method:**

Focusing on hippocampus and dorsolateral prefrontal cortex (dlPFC), we tested whether the association between regional grey matter volumes and self‐reported physical activity differs between women and men. We examined this interaction in five European cohorts from the Lifebrain consortium (*n* = 1,809; age range: 18 – 88 years). Effect sizes were first determined by linear models run separately for each cohort, then pooled across datasets in a random‐effects meta‐analysis.

**Result:**

Contrary to our hypotheses, there was no evidence of a relationship between physical activity and hippocampal or dlPFC volumes, nor was there a moderation by sex.

**Conclusion:**

Our null findings raise the question of whether self‐report questionnaires of physical activity, which commonly feature in big datasets, are sufficiently sensitive to capture a – presumably modest – association between physical activity levels and grey matter outcomes. We conclude that the reliance on self‐report questionnaires of physical activity is sub‐optimal for brain‐behaviour analyses.

